# Use of Handgrip Strength as a Health Indicator in Public Sector Workers: A Cross-Sectional Study

**DOI:** 10.3390/jfmk10020135

**Published:** 2025-04-17

**Authors:** Raynann Crislayne de Souza Borges, Mayara Bocchi, Joyce Cristina Freitas, Ana Karolina Assis Carvalho Silva, Luiz Fernando Gouvêa-e-Silva, David Michel de Oliveira, Eduardo Vignoto Fernandes

**Affiliations:** 1 Department of Physiotherapy, Federal University of Jataí, Jataí 75801-615, Brazil; crislayne_raynann@discente.ufj.edu.br; 2 Department of Medicine, Federal University of Jataí, Jataí 75801-615, Brazil; mayara.fernandes@ufj.edu.br; 3 Postgraduate Program in Bioscience and One Health, Federal University of Jataí, Jataí 75801-615, Brazil; joyce.freitas@discente.ufj.edu.br (J.C.F.); ana.karolina@discente.ufj.edu.br (A.K.A.C.S.); profdoliveira@ufj.edu.br (D.M.d.O.); 4 Laboratory of Human and Comparative Anatomy, Federal University of Jataí, Jataí 75801-615, Brazil; lfgouvea@ufj.edu.br; 5 Department of Physical Education, Federal University of Jataí, Jataí 75801-615, Brazil

**Keywords:** workplace health promotion, functional capacity evaluation, health promotion

## Abstract

**Objectives:** Handgrip strength (HGS) has been used as an important health indicator due to its ability to predict morbidity and mortality risks in various populations. The aim was to investigate the relationship between HGS and health parameters among public sector workers. **Methods:** A cross-sectional study was carried out, including the participation of 129 workers. The body composition, presence of chronic non-communicable diseases (CNCDs), physical activity level, and physical capacity (flexibility, HGS, and lower limb endurance) of the workers were evaluated. **Results:** The results showed that most of the employees, regardless of sex, work in the administrative sector, are aged 40 years or older, have chronic non-communicable diseases (CNCDs), and are physically inactive. The majority of women have a body mass index (BMI) within the normal range and are more flexible. On the other hand, men demonstrated greater lower limb endurance. Lower HGS was associated with a higher BMI, the presence of a CNCD, and worse lower limb endurance. A negative correlation was observed between HGS and age, BMI, and body fat. Conversely, a positive correlation was found between HGS and muscle mass, as well as with lower limb muscle endurance. **Conclusions:** It can be concluded that individuals with lower HGS are more likely to have a high BMI, develop a CNCD, and present worse lower muscular endurance. Additionally, HGS was found to be reduced in older individuals and/or those with higher levels of body fat, and to be higher in those with normal physical capacity and body composition.

## 1. Introduction

The preservation of workers’ health and well-being not only contributes to their personal development but is also a crucial component for optimizing productivity and efficiency at work [1]. However, this population group faces significant challenges related to work overload, high stress levels [2,3], and changes in habits, which directly impact their quality of life [4].

The lack of resources and infrastructure, professional devaluation, limited autonomy, physical overload, lack of cooperation among colleagues, reduced team sizes, and long working hours harm health and work performance [5]. Stressful factors at work can also trigger health conditions, such as gastrointestinal problems, anxiety, depression, alcohol dependency, sleep disorders, increased aggressiveness, and suicidal thoughts [6], that negatively impact workers’ health and can result in the onset of chronic non-communicable diseases (CNCDs), affecting productive capacity [7].

CNCDs, which include cardiovascular diseases, cancer, diabetes, and chronic respiratory diseases, constitute the main public health challenges globally [8]. In addition, poor working conditions are among the primary determinants of morbidities in administrative and healthcare sector employees [9,10]. It is recognized in the literature that many cases of morbidity and mortality are associated with unhealthy behaviors, such as physical inactivity [11]. These conditions worsen the disease state, as one morbidity can exacerbate another, due to their intrinsically similar mechanisms [12].

The complexity of CNCDs, combined with musculoskeletal disorders (MSDs), and their relationship with a stressful work environment require a multifaceted approach for effective occupational health management [1]. MSDs, also referred to as cumulative traumatic disorders, are diseases that affect workers from different occupational sectors. These pathologies can cause inflammation and degeneration, injuries that mainly affect the muscles, nerves, tendons, joints, and cartilage, resulting in pain and functional limitations, which are the occupational problems that cause the most harm to workers [13].

Handgrip strength (HGS) is a widely used tool, due to its ability to predict the risk of morbidity and mortality in various populations. This tool is used to evaluate the muscle strength of the upper limbs, specifically the forearm and hand, and is measured using a dynamometer. Furthermore, HGS has also been used to measure work capacity and the functional impact of injuries, and as a method for measuring and evaluating the progression of worker rehabilitation [14], acting as a health indicator related to cardiometabolic diseases, bone health, muscle strength and health, and nutritional status [15,16].

Studies indicate that lower HGS is positively associated with the risk of type 2 diabetes mellitus [17], arterial stiffness [18], cardiovascular disease [19], and stroke [20]. Other comorbidities have been related to lower muscle strength and, consequently, lower HGS, such as chronic kidney disease [21], cancers [22], depression, cognitive impairment, and poor mental health [23].

Individuals with lower HGS also have a higher risk of developing work-related musculoskeletal disorders, especially workers who adopt inadequate postures and work long hours [24]. In addition, the literature data indicate that a sedentary lifestyle, common among adults in work environments requiring prolonged sitting, is associated with a significant decrease in HGS and, consequently, in overall muscle strength [25].

Given the above, it is hypothesized that lower HGS is associated with poorer health indicators and functional capacity in public sector workers. In this sense, the objective of the present study was to investigate the relationship between HGS and health parameters in public sector workers, as conducting research on such indicators is essential for the implementation and improvement of institutional policies aimed at maintaining occupational health.

## 2. Materials and Methods

### 2.1. Type of Study, Site, and Sample

This is a cross-sectional study with a quantitative approach, carried out with employees in the administrative sector of the Municipal Health Department (MHD) in the city of Jataí, located in the southwest region of the state of Goiás, Brazil.

The sample size was calculated using the G*Power^®^ simulator, version 3.1.9.2 (Institute for Experimental Psychology in Düsseldorf, Germany), with type I and II errors defined as α = 0.05 and β = 0.05, respectively, to achieve an effect size of 0.30 or greater. Based on the sample size calculation, data collection was determined for 115 participants.

The 136 MHD employees were invited to participate in the assessments randomly and voluntarily. These assessments were linked to the extension project “Labor gymnastics for improving workers’ health and quality of life”, offered by the Federal University of Jataí (UFJ). In total, 129 employees agreed to participate in the assessments.

The study was conducted in accordance with the Declaration of Helsinki. To authorize the use of the project data, the study was approved by the UFJ Research Ethics Committee (opinion: 6.625.537; CAAE 75454123.2.0000.0187) on 26 January 2024. After approval by the committee, the project was registered with the Brazilian Clinical Trials Registry Platform (ReBEC) under the following number: RBR-10v25rm5 (https://ensaiosclinicos.gov.br/rg/RBR-10v25rm5), accessed on 25 January 2025. Participants were informed and their verbal consent was obtained.

### 2.2. Inclusion and Exclusion Criteria

The inclusion criteria were as follows: being an employee of the Jataí MHD, being 18 years of age or older, and not having physical limitations and cognitive limitations that prevent the individual from performing the physical tests and answering the questionnaires. The exclusion criteria were as follows: not participating in all stages of the physical assessment and not answering all questionnaires.

### 2.3. Experimental Design

The experimental design included the following steps: initially, the participants completed questionnaires about the role they perform (administrative sector or healthcare professional), sex, age, presence of CNCD, and physical activity level (PAL). Next, body composition assessments were conducted (body mass, height, body mass index (BMI), triceps skinfolds, percentage of fat, and muscle mass). Finally, the functional tests were performed (HGS, flexibility, and lower limb endurance).

### 2.4. Procedures

#### 2.4.1. Physical Activity Level (PAL)

PAL was assessed using the International Physical Activity Questionnaire—Short Form (IPAQ-SF). The IPAQ-SF classifies participants as very active, active, insufficiently active, or sedentary [26]. To present the data, in the current study, individuals were classified as active (very active + active) or inactive (insufficiently active + sedentary), as proposed by [27]. It is worth mentioning that the evaluators who participated in the data collections had training in Physical Education, with over 15 years of experience in carrying out anthropometric assessments and physical tests. Regarding data collection, all employees were evaluated in the afternoon, that is, between 1:00 p.m. and 5:00 p.m. In addition, the researchers informed the participants not to perform moderate to vigorous physical activities 48 h before the tests.

#### 2.4.2. Body Mass and Height

Body mass was measured using a calibrated analogue scale (Welmy^®^, Welmy Indústria e Comércio Ltda, Santa Bárbara d’Oeste, Brazil), accurate to 100 g. Height was measured using a stadiometer attached to the scale, accurate to 0.5 cm. The body mass index (BMI) was calculated by dividing the body mass (kg) by height in meters squared. BMI classification was in accordance with those defined by the World Health Organization (WHO) [28].

#### 2.4.3. Body Composition

Body composition was measured using an Omron bioimpedance scale (HBF-514C), which provided the percentage of fat and muscle mass. Equipment was validated by dual-energy X-ray absorptiometry, demonstrating a correction coefficient of 0.91 [29]. Waist and hip circumference were measured using a Sanny^®^ (American Medical do Brasil Ltda, Sao Bernardo do Campo, Brazil) anthropometric tape, and the waist-to-hip ratio was calculated. The right triceps skinfold was measured using a Cescorf^®^ (Cescorf Equipamentos para Esporte Ltda, Porto Alegre, Brazil) adipometer, accurate to 0.1 mm, and the average of three measurements was used for the results.

#### 2.4.4. Handgrip Strength (HGS)

HGS (dominant limb) was measured using an analogue dynamometer (Saehan^®^, Saehan Corporation, Gwangju, Republic of Korea) accuracy of 2 kg, with the forearm in 90° flexion, and the highest value from three attempts (30 s interval between attempts) was adopted as the result [30]. The absolute strength values were divided by the body mass to find the relative HGS, as proposed in the study by [16]. The relative HGS classification was considered according to the quartile; that is, the relative HGS values in the 1st quartile (25%) were considered as lower HGS, and the relative HGS values in the other quartiles (>25%) as higher HGS [30].

#### 2.4.5. Flexibility

Flexibility of the hamstring muscles and lower back was measured using the Wells Bench sit and reach test [31]. The test was carried out using a box measuring 30 cm long × 29 cm high × 30 cm wide, with a 27 cm scale at the top. The zero point is at the end closest to the subject and the 27 cm point coincides with the footrest. The subject removed their shoes and, in a sitting position, placed the soles of their feet on the box with their knees extended. With shoulders flexed, elbows extended, and hands supinated, the participant flexed their hips and touched the furthest point possible on the scale with their hands [32].

#### 2.4.6. Lower Limb Endurance

Lower limb endurance was measured using the 30 s sit to stand test (SST). This test consists of sitting down and standing up from a chair as many times as possible in 30 s [33].

### 2.5. Statistical Analysis

Initially, the data were subjected to the Kolmogorov–Smirnov normality test. The associations between HGS and BMI, CNCDs, and SST were evaluated using the Fisher’s exact test. The Pearson’s r test was used to correlate relative HGS with participants’ age, body composition, and SST. It is pertinent to note that the categorical distribution of age, flexibility, and SST was consistent with the mean values observed among the participants. Regarding the BMI distribution, we adhered to the WHO classification, wherein a BMI ≥ 25 kg/m^2^ is indicative of overweight, and a BMI < 25 kg/m^2^ is classified as normal weight. The statistical program used was GraphPad Prism 9.5.1 and the minimum significance level adopted was *p* < 0.05.

## 3. Results

Initially, all 136 employees of the SMS were invited to participate in the study, of whom 5 declined to participate in the data collection and 2 were not eligible based on the inclusion criteria. Thus, 129 people were evaluated, 98 females, with an average age of 43.6 ± 10.8 years, and 31 males, with an average age of 43.3 ± 10.0 years. Regarding height, body mass, and BMI, the female participants presented the following average values: 1.60 ± 0.06 m, 72.1 ± 18.1 kg, and 27.8 ± 6.4 kg/m^2^, respectively; and the male participants: 1.72 ± 0.06 m, 87.8 ± 15.8 kg, and 29.5 ± 4.7 kg/m^2^, respectively.

Table 1 presents the characteristics of the workers. It was observed that, regardless of sex, most employees work in the administrative sector, are over 40 years of age, have a CNCD, and are physically inactive. On the other hand, when analyzed according to sex, most women showed better BMI levels (<25 kg/m^2^; 57.1%) and flexibility (≥25 cm; 57.7%), while men demonstrated greater lower limb endurance, as evidenced by the SST (≥25 repetitions in 30 s; 64.5%).

Table 2 presents the associations between lower and higher relative strength with BMI, CNCDs, and SST. An odds ratio of 3.2 times was found for people with lower strength to have a higher BMI (*p* = 0.01) and a CNCD (*p* = 0.01). Regarding muscle endurance, evaluated by the SST, people with lower strength presented a 6.2-times-higher odds ratio of having worse lower limb endurance (*p* = 0.001). No associations were found with function, age, PAL, and flexibility (*p* > 0.05).

**Figure 1:**
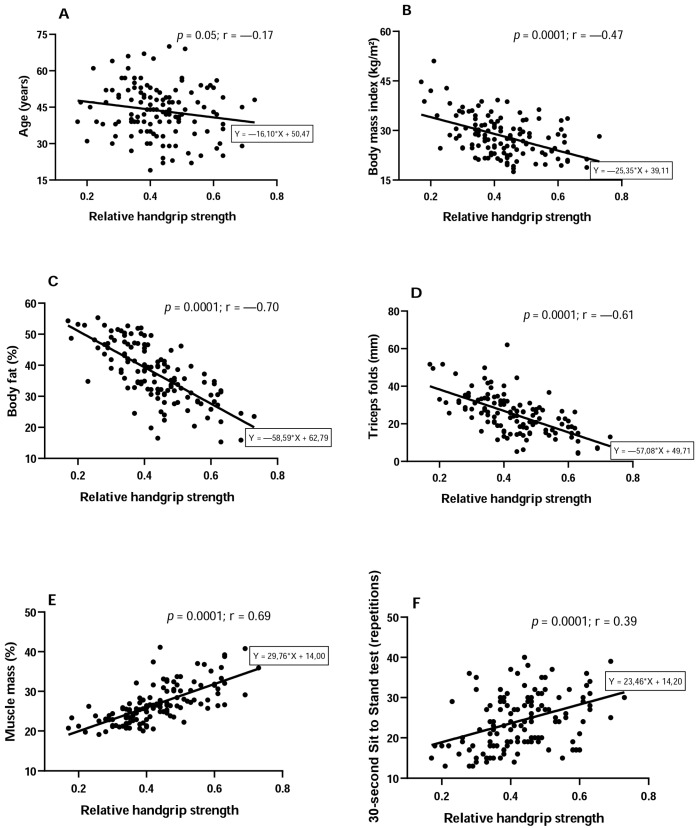
Correlations between relative handgrip strength and age (**A**), body mass index (**B**), body fat (**C**), triceps skinfold (**D**), muscle mass (**E**), and the 30 s sit to stand test (**F**).

Figure 1 presents the correlations between relative HGS and age, body composition, and muscle endurance of the employees. An inverse correlation was observed between age (Figure 1A: *p* = 0.05; r = −0.17), BMI (Figure 1B, *p* = 0.0001; r = −0.47), fat percentage (Figure 1C: *p* = 0.0001; r = −0.70), and triceps skinfold (Figure 1D: *p* = 0.0001; r = −0.61) in relation to relative HGS. On the other hand, there was a positive correlation between relative HGS and muscle mass (Figure 1E: *p* = 0.0001; r = 0.69) and SST (Figure 1F: *p* = 0.0001; r = 0.39). Regarding flexibility, no correlation was found with relative HGS (*p* = 0.52, r = 0.05).

## 4. Discussion

According to the data obtained in the present study, it was observed that the majority of the MHD workers evaluated, regardless of sex, work in the administrative sector, are over 40 years of age, have a CNCD, and are physically inactive. These findings are consistent with the literature that identified a similar age group profile to that found in the present study [34]. Additionally, among workers in this age group, it is common to find individuals with health patterns similar to those observed in the present research, namely, a high prevalence of CNCDs and low PAL, as indicated by [35,36].

Administrative sector workers often lead a physically inactive lifestyle, with short or no breaks during the workday [37]. This hypokinetic behavior leads to low energy expenditure, resulting in obesity, insulin resistance, and chronic inflammation [38]. Studies indicate that long periods of sitting are associated with physical inactivity and/or sedentary behavior [39,40,41] and increase one’s predisposition to the development of CNCDs [42]. Additionally, factors such as being female, having a dual workload, advanced age, and lower educational levels are also associated with an increase in physical inactivity [43].

In the current study, the BMI values presented by both sexes were greater than 25 kg/m^2^, which characterizes overweight [44]. The main factors that can influence weight gain among professionals who work long hours are hormonal dysregulation, the consumption of ultra-processed foods, and low rates of physical activity [45].

When considering flexibility and muscle endurance, it was observed that women are more flexible, while men demonstrated greater lower limb endurance. These results confirm the differences between sexes in physical abilities, as documented in the literature, where it is commonly observed that women tend to have greater flexibility and men greater strength and muscular endurance, which is multifactorial in nature. These factors may include sex differences in height, body weight, bone and arm length, testosterone levels, muscle mass, agonist muscle voluntary activation, antagonist muscle coactivation, and participation in physical activities during childhood [46]. Higher testosterone levels in males promote an increase in muscle mass, which contributes to strength gain, while women, in turn, have higher levels of estrogen and relaxin compared to men, thus contributing to the elasticity of connective tissues and joint range of motion, resulting in greater flexibility [47].

In the present study, an inverse correlation was also observed between age and relative HGS. As age advances, there is progressive loss of muscle mass and strength, leading to a decline in functional capacity and an increased risk of developing a CNCD. Age-related muscle loss is attributed to a disruption in the regulation of skeletal muscle protein turnover, resulting in an imbalance between muscle protein synthesis and degradation [48]. Muscle strength is necessary for performing daily activities and is considered a marker of overall health [49]. Similarly, in the study by [50], a relationship was observed between HGS and physical capacity in healthy individuals. Thus, HGS may be a factor that contributes to better functionality outcomes in individuals.

The association between lower HGS with elevated BMI and the prevalence of individuals with a CNCD found in the present study are consistent with the data from the literature. A 22-year longitudinal study conducted in Finland, involving 963 people of both sexes aged 30 to 73, found that physically strenuous work, excess adipose tissue, smoking, cardiovascular disease, hypertension, diabetes mellitus, and asthma were predictors of muscle strength decline in workers [51]. The literature data also point to an association between an inadequate lifestyle (hypercaloric nutrition and sedentary behavior) and obesity, conditions related to the increase in pro-inflammatory mediators [52]. Age-associated adiposity exerts an adverse influence on inflammatory markers, exacerbating the infiltration of adipose tissue into skeletal muscles, which compromises structural integrity and the ability to generate strength [53,54].

In a study involving 1395 people of both sexes, it was observed that individuals with a CNCD had lower HGS, and higher BMI levels, abdominal circumference, and fat mass compared to the control group. These results were also reflected in the sum of skinfold thickness and the body fat percentage of the group with lower HGS [55]. These findings align with the data from the present study. However, the current study did not find an association between relative HGS and variables such as age and flexibility. This suggests that lower relative HGS has a more direct impact on body composition, the presence of a CNCD, and lower limb muscle endurance, while other variables may be influenced by factors such as lifestyle, genetics, and individual characteristics [56].

Lower HGS has been identified as a marker of musculoskeletal fitness frailty [57,58]. In a study involving 18,708 participants, it was found that factors such as low mobility, the presence of pain, and behavioral disorders were associated with lower HGS in both sexes [59]. Additionally, another study revealed that individuals with lower HGS (<28 kg for men and <18 kg for women) had lower SST performance [60]. These results reinforce the findings of the present study, where a positive correlation was observed between relative HGS, muscle mass, and performance in the SST.

The literature indicates that forearm circumference, hand length, and hand width are also reliable predictors of HGS. Furthermore, factors such as lifestyle habits (level of physical activity, nutritional aspects, and alcohol and tobacco consumption) and the employee’s type of work should also be considered for a more accurate HGS assessment [14]. It is important to note that muscle strength is just one component of health-related physical fitness. Therefore, in clinical practice, it is crucial that we monitor an individual’s overall fitness using other parameters like muscle endurance, flexibility, and body composition. Collectively, these factors provide valuable input for companies to develop intervention policies aimed at employee well-being [61].

Although the current study presents some limitations, such as the sample being from a single company and the absence of control over confounding variables like diet and the daily life habits of the participants, these did not compromise the results. A sample size calculation was performed, and the number of people evaluated was representative for the study, which allowed for important inferences about the relationship between HGS and health indicators.

## 5. Conclusions

This study presents indicators that individuals with lower HGS could be more likely to have a higher BMI, develop a CNCD, and present worse lower muscular endurance. Additionally, HGS was found to be lower in people who are older and/or have higher levels of body fat, and higher in individuals who present physical capacity and body composition parameters within normal ranges.

These results reinforce the role of HGS as a relevant health indicator among workers, highlighting its usefulness not only in the early detection of risk factors but also in promoting the ongoing general monitoring of physical condition. The use of HGS can, thus guide more effective preventive interventions, helping in the early identification of physical imbalances and potential health risks, such as obesity, sarcopenia, and cardiovascular diseases. This suggests that incorporating an HGS assessment into occupational exams and workplace wellness programs can contribute to improving the quality of life for workers.

As a strategy for worker intervention and care, it is suggested that strength assessments be conducted periodically within the company, at least every 12 weeks, alongside habit modifications such as incorporating breaks for physical activity during working hours, as well as postural care, i.e., maintaining adequate ergonomics. These measures should be reinforced by companies through policies that prioritize worker health.

## Figures and Tables

**Table 1:** Functional characteristics, age, and body composition of the employees.

Variables	Male	Female
	*n* (%)	*n* (%)
*Function*		
Administrative sector	24 (77.4)	63 (64.3)
Healthcare professional	07 (22.6)	35 (35.7)
*Age (years)*		
<40	11 (35.5)	36 (36.7)
≥40	20 (64.5)	62 (63.3)
*CNCD*		
Yes	17 (54.8)	63 (64.3)
No	14 (45.2)	35 (35.7)
*PAL*		
Active	12 (38.7)	47 (48.0)
Inactive	19 (61.3)	5 (52.0)
*BMI (kg/m^2^)*		
<25	06 (19.4)	56 (57.1)
≥25	25 (80.6)	42 (42.9)
*Flexibility (cm)*		
<25	18 (56.2)	41 (42.3)
≥25	14 (43.8)	56 (57.7)
*SST (repetitions)*		
<25	11 (35.5)	58 (59.2)
≥25	20 (64.5)	40 (40.8)

Legend: CNCD, chronic non-communicable disease; PAL, physical activity level; BMI, body mass index; SST, 30 s sit to stand test. Data expressed in absolute (n) and relative values (%).

**Table 2:** Association between relative strength and body composition, presence of chronic non-communicable diseases, and lower limb endurance in employees.

Variables	HGS Relative		*p*	OR (95% CI)
	Lower	Higher		
	*n* (%)	*n* (%)		
*BMI (kg/m^2^)*				
≥25	29 (82.9)	56 (59.6)	0.01	3.2 (1.29–8.40)
<25	06 (17.1)	38 (40.4)		
*CNCD*				
Yes	28 (80.0)	52 (55.3)	0.01	3.2 (1.24–8.11)
No	07 (20.0)	42 (44.7)		
*SST (repetitions)*				
<25	29 (82.9)	41 (43.6)	0.001	6.2 (2.47–15.92)
≥25	06 (17.1)	53 (56.4)		

Legend: BMI, body mass index; CNCD, chronic non-communicable disease; SST, 30 s sit to stand test. OR, odds ratio; 95% CI, 95% confidence interval.

## Data Availability

The data are available upon reasonable request.
